# Knowledge, Attitudes, and Practice among Women and Doctors Concerning the Use of Folic Acid

**DOI:** 10.5402/2011/946041

**Published:** 2010-10-03

**Authors:** Eithan Auriel, Aya Biderman, Ilana Belmaker, Tamar Freud, Roni Peleg

**Affiliations:** ^1^Department of Family Medicine, Siaal Research Center for Family Medicine and Primary Care, Faculty of the Health Sciences, Ben-Gurion University of the Negev, P.O. Box 653, Beer-Sheva 84105, Israel; ^2^Clalit Health Services, Southern District The Negev Mall, Eli Choen Junction, Beer-Sheva 84105, Israel; ^3^The Israel Ministry of Health, Southern Region, Hatikva 4 St, P.O. Box 10050, Government Campus, Beer- Sheva 84170, Israel

## Abstract

*Background and Objective*. Daily folic acid intake, prior to conception and in early pregnancy, significantly reduces neural tube defects (NTDs). We compared folic acid consumption among Jewish and Bedouin women and the recommendations of family physicians and gynecologists. *Methods*. We compared 64 Muslim Bedouin women and 65 Jewish women. We also compared 39 gynecologists and 60 family physicians. *Results*. Fifty-one Jewish women (78.5%) took folic acid during pregnancy, but only seven (10.8%) before conception. Sixty Bedouin women (93.75%) took folic acid during pregnancy, but only four (6.25%) before conception (*P* < .05). Five Jewish women (7.7%) and two Bedouin women (3.1%) took folic acid three months before conception. Thirty-three gynecologists (87%) recommend preconception folic acid compared with thirty-six family physicians (60%) (*P* < .05). *Conclusions*. The majority of women use folic acid during pregnancy, but only few do so to prevent NTDs. There is a significant difference between doctors' recommendations and actual practice.

## 1. Introduction

Neural tube defects (NTDs), including spina bifida, anencephaly, and encephalocele, are among the most common birth defects and cause morbidity and mortality among fetuses and babies [1].

The incidence of NTDs in the United States is approximately one per 1,000 pregnancies. Approximately one third of 4,000 pregnancies with NTDs end with spontaneous or induced abortion, so there are about 2,500 live births with NTD annually in the US [2]. There is a significant geographical variation in the rate of NTDs [3]. In the years 1999-2000 there were at least 394 cases of isolated NTD in Israel, with a nearly fourfold incidence among non-Jews compared to Jews [4].

The embryonic basis of NTD is not fully understood, but a common abnormality is lack of closure of the neural tube [3]. The normal development and closure of the neural tube are completed by the 25th day after fertilization.

The mortality rate among newborns with spina bifida is 10% in the United States, but nearly 100% in China [3]. Among surviving babies there is a high rate of neurological complications such as paralysis, hydrocephalus, Arnold Chiari type II and syringomyelia [3]. The known risk factors for the development of NTD are low socioeconomical class, maternal diabetes, antiepileptic drugs, fever in the early stage of pregnancy, and obesity [3]. 

In families with a history of NTD, there is a 2%-3% risk of NTD in future pregnancies. The association between folic acid supplementation and reduced rates of NTD is well established [5]. A study among 130,142 Chinese women between 1993–1995 demonstrated a reduction in the risk for NTD of up to 85% in women who took 0.4 mg/day of folic acid prior to conception and during the first trimester [6] and other studies have provided additional evidence of this beneficial effect [1].

Folic acid is absorbed in the proximal part of the small intestine and stored in the liver and CSF. About 4-5 microgram is excreted daily in the urine. The mean dietary intake of folic acid is about 0.1-0.2 mg a day. Folic acid is present in food products such as liver, kidney, nuts, yeast, and green vegetables. It is reduced in the body to tetrahydrofolate, a coenzyme for many metabolic reactions including DNA synthesis and amino acid conversion. Folate deficiency can result in megaloblastic anemia. The recommended daily dose of folic acid to prevent megaloblastic acid in pregnancy is 0.2–0.5 mg/day. Folic acid supplementation is well tolerated, although gastrointestinal side effects may occur. Folic acid also plays an important role in the prevention of arteriosclerosis [5].

The Israel Ministry of Heath recommends a daily intake of folic acid of 0.4 mg/day three months before conception and throughout the first trimester. Women who previously conceived an infant with NTD should take 0.4 mg/day of folic acid throughout fertility and 4-5 mg/day beginning one month before conception and throughout the first trimester [7].

In the present study we assessed the consumption of folic acid for the prevention of NTDs among Jewish and Muslim Bedouin women in southern Israel and analyzed possible correlations with the recommendations of family physicians and gynecologists.

The Jewish sector in Beer-Sheva is classified as low-middle class. The Bedouin sector in southern Israel is of a very low socioeconomic status, with many living in small stone houses, wooden or tin huts, or even tents. A considerable percentage of this population is supported by welfare and has only a few years of formal education [8].

## 2. Methods

### 2.1. Study Group

The study population included 65 pregnant Jewish women from the city of Beer-Sheva, 64 pregnant Bedouin women from the Negev desert region of southern Israel, 39 gynecologists (specialists and residents) from the Soroka University Medical Center in Beer-Sheva and 60 family physicians (specialists and residents) from the Negev region. The Helsinki Committee of the Soroka Medical Center in Beer-Sheva and the Israel Ministry of Health approved the study.

The interviews in the Jewish sector were held in three clinics in Beer-Sheva that represent populations of different socioeconomic status, as determined by the Israel Ministry of Health. The pregnant women were interviewed personally by the first author (EF) upon arrival for routine check-up at the “Mother and Child Health Clinics” after consenting to be interviewed and after it was clear that they had not been interviewed previously. Women visiting the clinic for the first time and women with a history of congenital anomaly in a previous pregnancy were excluded.

The interviews in the Bedouin sector were held at a clinic in a Bedouin village and in a Beer-Sheva clinic that serves Bedouins living in rural areas outside established villages. In those clinics we used a questionnaire that was translated into Arabic with the aid of an interpreter.

The doctors completed the questionnaires at work or while attending continuing medical education courses at the university.

### 2.2. The Study Tool

An anonymous questionnaire that included questions on demographic background, knowledge and use of folic acid was administered to the pregnant women. A second anonymous questionnaire that included questions on demographic background, knowledge, attitudes, and recommendations regarding folic acid use was given to the doctors.

### 2.3. Statistical Methods

The study data were organized with the Excel software program and processed using the SSPS statistical package. Group differences were compared by the *χ* ^2^ test and statistical significance was set at *P* < .05.

## 3. Results

### 3.1. Pregnant Women

The mean maternal age among 65 Jewish women was 28.6 ± 4.9 years (range 20–39) and 27.9 ± 5.6 among Bedouin women (range 17–40 years). Four Bedouin women and nine Jewish women refused to participate. The mean number of children per woman was significantly higher in the Bedouin sector. The women from the Jewish sector were significantly more educated than the women from the Bedouin sector.  Table 1 presents the demographic background of the Jewish and Bedouin women. 

Fifty-one Jewish women (78.5%) reported taking folic acid during pregnancy, but only seven (10.8%) began prior to conception. Sixty Bedouin women (93.75%) reported taking folic acid during pregnancy, but only four (6.25%) began prior to conception (*P* < .05) (Table 2). Only five Jewish women (7.7%) and two Bedouin women (3.1%) began taking folic acid three months before conception as recommended by the Israel Ministry of Health. Sixty-one Jewish women (94%) and all of the Bedouin women said that they had received these recommendations or knew about them. Seven of the ten Jewish women and three of the four Bedouin women who refrained from taking folic acid cited as the reason previous side effects such as nausea, weakness and dizziness that they attributed to it. Twenty-eight Jewish women (43.1%) 18 Bedouin women (28.1%) believed that folic acid could cause side effects. Twenty three Jewish women (35.4%) and four Bedouin women (6.3%) knew that folic acid could prevent NTDs. Twenty of the Bedouin women knew that taking folic acid could “help” the fetus, but could not define specific health benefits. 

Only two Jewish women (3.1%) and 10 Bedouin women (15.6%) had received and read the Ministry of Health publication on prevention of NTDs, which is available at the Mother and Child Clinics.

### 3.2. Doctors

The study was conducted among 60 family physicians (54% women, mean age 38.9 ± 7.6 years, range 29–56) and 39 gynecologists (26% women, mean age 39.5 ± 8.5 years, range 29–59).  Table 3 presents the demographic background of the doctors. More gynecologists than family physicians were department or unit heads, while more family physicians were still in various stages of professional training (residents).

Thirty-three gynecologists (87.9%) and 36 family physicians (60%) claim to always recommend folic acid on preconception examination. All the gynecologists and 55 family physicians (92%) reported that they almost always or always recommend folic acid supplementation at this stage (*P* < .05). Thirty-three gynecologists (84%) and 44 family physicians (73%) always recommend folic acid supplementation during pregnancy.  Table 4 presents the physician recommendations before and during pregnancy. Only 35 of 56 family physicians (62.5%) and 28 of 39 gynecologists (71.8%) recommended the correct daily dose of 0.4 mg. Eight family physicians (32.1%) and nine gynecologists (21.3%) recommended a high daily dose of 5 mg.

Seventeen of 37 gynecologists (45.9%) and 32 of 57 family physicians (56.1%) believe that more than 30% of the women actually begin to take folic acid prior to conception.

## 4. Discussion

Folic acid supplementation before and during pregnancy is an inexpensive, simple strategy that can significantly reduce the incidence of NTD, a very severe malformation. The average lifetime cost of a baby with NTD is estimated at about $294,000 [3]. There is evidence that folic acid can also prevent other types of congenital anomalies [1]. Folic acid treatment before and during pregnancy is recommended by the Israel and the United States Ministries of Health and by the American Academy of Pediatrics [2, 9].

The results of the present study demonstrate that despite the clear recommendations, only 10.8% of Jewish women and 6.25% of Bedouin women take folic acid prior to conception, although 78.5% of the Jewish women and 93.75% of the Bedouin women said they took folic acid at some time during pregnancy. 

The issue of folic acid supplementation prior to conception has been studied in many countries. In British Columbia [10] 49.4% of 1,004 took folic acid prior to conception. In Puerto Rico [11] 31.5% of 479 women took folic acid appropriately. In Ireland [12] 16% of 300 women took folic acid prior to conception. In a previous Israeli study conducted in 1999 [13] only 2.8% of 920 women took folic acid before conception.

In the present study we found that although more Bedouin women take folic acid during pregnancy than Jewish women, fewer Bedouin women begin taking it prior to conception. Since a relatively high rate of Jewish and Bedouin women take folic acid during pregnancy, even if inappropriately, the potential exists for better adherence to recommendations with a concomitant reduction in the rate of NTDs.

The rate of NTDs is almost fourfold among Bedouins and other non-Jewish populations compared to Jews [4]. The reasons for this finding include consanguineous marriage among Bedouins, differences in nutrition, and religious prohibition of abortions. Special intervention plans should be developed for each population based on its cultural and socioeconomic characteristics. 

A minority of women who did not take folic acid during pregnancy despite recommendations cited fear of side effects that they attributed to its use during previous pregnancies. Even the high dose of folic acid (5 mg) prescribed by 32.1% of the family physicians and 23.1% of gynecologists is unlikely to explain the reported side effects, since these side effects are rare even at this higher dose. It is possible that some of the women used this explanation as an excuse because they were embarrassed that they did not take folic acid.

There is a significant difference between doctors' reports and the actual degree to which women took folic acid prior to conception. All the gynecologists and 92% of the family physicians in the study said that they always/almost always recommend taking folic acid prior to conception, but only 10.8% of the Jewish women and 6.25% of the Bedouin women reported taking folic acid at this stage. On the other hand, there is correspondence between the doctors' reports of recommendations and the pregnant women's reports of folic acid use during pregnancy.

Despite the study's small sample size the data demonstrate that only a small minority of women use folic acid appropriately. Since folic acid supplementation is the only way to provide 4-5 mg of folic acid daily it is important to increase the public's awareness of this requirement. An intervention plan should include three basic strategies [14]: diet, folic acid supplementation, and food fortification. According to a survey conducted in the year 2000 the mean daily dietary intake of folic acid is 213.8 ± 181.8 micrograms among Jewish women and 302.0 ± 226.9 micrograms among Bedouin women (unpublished data). This difference may be due to the relatively high intake of fresh vegetables by Bedouins.

Our study demonstrates that only a small minority of the women received and read the Ministry of Health's publication on this issue. Other studies have reported that popular newspapers and the electronic media are more effective than printed material disseminated by health authorities [9].

## 5. Conclusions

It is important to train health system workers in the community to recommend folic acid before, and not only after, conception. It is important for physicians to encourage a preconception examination in which counseling and recommendations for folic acid supplementation are provided. Special attention should be given to family physicians since, according to the results of the present study, only 60% always recommend folic acid prior to conception.

## Figures and Tables

**Table 1:** The demographic characteristics of the jewish and bedouin women.

Variable	Jews (*n* = 65)	Bedouins (*n* = 64)	*P*
Age [*N* (%)]			
17–20	2 (3)	7 (11)	NS
21–30	41 (63)	36 (57)	
>30	22 (34)	20 (32)	
Mean ± SD	28.6 ± 4.9	27.9 ± 5.6	

Present pregnancy			
First to third	48 (74)	32 (50)	<.05
Fourth or more	17 (26)	32 (50)	
Mean ± SD	2.6 ± 1.6	4.0 ± 3.0	

Children			
0	26 (40)	14 (22)	<.05
1–3	34 (52)	29 (46)	
4+	5 (8)	20 (32)	
Mean ± SD	1.2 ± 1.3	2.8 ± 2.7	

Education			
None	1 (1.5)	14 (22)	<.0001
Elementary	1 (1.5)	20 (31)	
High school	63 (97)	30 (47)	

**Table 2:** Comparison of consumption of folic acid, by sector and time during pregnancy.

Relation to pregnancy	Bedouins *N* (%)	Jews *N* (%)	*P*
Before and during	4 (6.25)	7 (10.8)	<.05
Only during	56 (87.5)	44 (67.7)	
Never	4 (6.25)	14 (21.5)	

**Table 3:** Comparison of gynecologists and family physicians.

Variable	Gynecologists (*n* = 39)	Family physicians (*n* = 60)	*P*
Sex			
Male	28 (74)	26 (46)	<.05
Female	10 (26)	31 (54)	

Age (yrs)			
<40	20 (62.5)	30 (64)	NS
≥40	12 (37.5)	17 (36)	
Mean ± SD	39.5 ± 8.5	38.8 ± 7.6	

Years as physician			
<10	19 (49)	30 (55)	NS
≥10	20 (51)	24 (45)	
Mean ± SD	13.7 ± 11.3	11.2 ± 7.9	

Position			
Department head	6 (15)	1 (2)	<.01
Other specialists	17 (43)	20 (33)	
Resident	16 (42)	39 (65)	

**Table 4:** Comparison of gynecologists and family physicians as to recommendations to take folic acid prior to conception and during pregnancy.

Extent of recommendation	Gynecologists *N* (%)	Family physicians *N* (%)	*P*
Prior to conception			
Always	33 (87)	36 (60)	<.05
Almost always	5 (13)	19 (32)	
Rarely	—	5 (8)	

During pregnancy			
Always	33 (84)	44 (73)	NS
Almost always	5 (13)	14 (23)	
Rarely	1 (3)	2 (4)	
